# Doctor—patient relationship when dealing with individuals with craniovertebral anomalies

**DOI:** 10.4103/0974-8237.65475

**Published:** 2010

**Authors:** Sunil K Pandya

**Affiliations:** Department of Neurosurgery, Jaslok Hospital and Research Centre, Dr. G. V. Deshmukh Marg, Mumbai 400 026, India

*'…Day and night, however thou mayest be engaged, thou shalt endeavour for the relief of patients with all thy heart and soul. Thou shalt not desert or injure thy patient even for the sake of thy life…*' –

Caraka [1]

## BACKGROUND

The relationship between a doctor and his patient is manifestly unequal, in favor of the doctor.

The patient may be in pain, have difficulty walking and working, and is anxious about worsening neurological symptoms. He is ignorant on matters medical. He is afraid of his illness and fears the worst consequences.

He comes seeking help, guidance, relief, and, if possible, cure from the doctor. He places his life in the doctor's hands.

Under these circumstances, the principle of equity demands that the rights of the patient take precedence over those of the doctor.

When surgical treatment is indicated for the patient with a craniovertebral anomaly, he needs special reassurance as an operation in the vicinity of the medullospinal junction, as with an operation in the neighborhood of the brainstem, carries risks to life, consciousness, breathing, limbs, and control over the passage of excreta.

The situation is especially worrisome when the craniovertebral anomaly has been accidentally discovered, the patient being free from symptoms from it.

In countries such as India, where the poor, deprived, and illiterate abound, the responsibilities of the doctor are even greater.

## SCIENTIFIC CONSIDERATIONS

The delicate balancing of the skull and its contents on the first and second cervical vertebrae evokes admiration. Over two kilograms are perched on a thin ring of bone that swivels around a pivot less than a centimeter in diameter. The arrangement of bones, ligaments, and muscles permits a wide range of movements that include nodding, turning, and tilting the head from side to side, looking up or down.

The passage of the medullospinal junction from the relatively large volume of the posterior fossa into the narrow confines of the spinal canal is also catered to marvellously. The smooth backward curve of the distal clivus and the oft-capacious cisterna magna provide adequate room. The cerebellum, within the confines of its fossa, stays well away from the lower medulla.

Abnormalities at the craniovertebral region reduce the available room and lead to compression and deformity of the crucial descending and ascending tracts and grey matter within the medullospinal junction. In doing so, they may produce a variety of neurological deficits.

Congenital dislocations of the atlas form an interesting category of craniovertebral anomalies. They may produce no pain and, at times, no symptoms whatsoever, being discovered accidentally on x-ray films of the upper neck. Atlanto-axial dislocations in patients with the mucopolysaccharidoses such as Morquio's disease are often overshadowed by the crippling physical deformities in chest and limbs and malfunctioning hearts. Likewise atlanto-axial dislocation may be detected late in the course of alcaptonuria, abnormalities of the lumbar spine, and in knee joints having taken center stage, by which time the medullospinal junction has already been damaged.

## MEDICAL RECORDS

"We should keep clear, up to date, detailed, and accurate clinical records, with full descriptions of operative procedures, significant medical events, medical management, the names of other caregivers, and summaries of important discussions with patients and family members." [2] (World Federation of Neurosurgical Societies 2008).

Such records, meticulously maintained and preserved, serve many purposes. They provide full details on all aspects of the patient's illness and treatment that can prove invaluable on subsequent visits by the patient to the clinic and even more so in the event the patient has to be readmitted for further care. A copy of the summary of findings and treatment, provided to the patient, can serve as a guide to other physicians who may be called upon to treat the patient at any stage, especially if the need arises in a foreign country. Finally, the medical record is the chief evidence that will be relied upon by the judge in court during any legal hearing.

The medical record is a confidential document and may be released to an outsider only after the patient has given express permission for such release. The World Federation of Neurosurgical Societies (2008) lists circumstances under which this confidentiality can be breached:

- "When patients may be entering an activity or employment for which they are unfit, and where they may pose a danger to others." In this case we should attempt to dissuade the patient, and attempt to obtain permission for disclosure when feasible.
- "When disclosure is a legal requirement," such as notification of specific disease.
- "When disclosure is required by a court of law."

To this may be added one more circumstance – when the patient is likely to harm or kill another. This exception to the rule of confidentiality was brought into sharp focus by the case Vitaly Tarasoff *et al*., Plaintiffs and Appellants, v. The Regents of the University of California *et al*., Defendants and Respondents. S.F. 23042. Supreme Court of California, July 1, 1976. [3] On October 27, 1969, Prosenjit Poddar killed Tatiana Tarasoff. Two months earlier Poddar confided his intention to kill Tatiana to Dr. Lawrence Moore, a psychologist employed by the Cowell Memorial Hospital at the University of California at Berkeley. The campus police briefly detained Poddar, but released him when he appeared rational. Dr. Harvey Powelson, Moore's superior, then directed that no further action be taken to detain Poddar. No one warned Tatiana of her peril. The court held that once a psychologist knows that a client poses a danger of violence to another, the psychologist has a duty to exercise reasonable care to protect the foreseeable victim of that danger. Discharging the duty may mean warning the potential victim.

While the Tarasoff decision is unlikely to apply to most patients with craniovertebral abnormalities, it is worth bearing in mind.

## THE SYMPTOMATIC PATIENT

Aware of the acute or progressive symptoms relating to the neck or limbs, the patient has more than an inkling of what is in store. When consultation is sought at a late stage of the illness, the considerable physical handicap makes the patient accept treatment with alacrity. It is easier to accept risky treatment when one is already in a desperate situation.

Even so, it remains the physician's duty to adhere strictly to the principles of medical ethics. Of these, *primum, non nocere*[4] must be paramount.

The patient must be informed of the nature of his illness, gravity of significance of the physical findings, and those on investigations such as X-ray films and magnetic resonance imaging. It is only when these are understood that the patient can offer his own views on proposed therapy and consent to what the physician proposes. The role of informed consent cannot be overemphasized here.

It is important to ensure that the information is not merely through printed sheets even though they may carry facts stated simply and in the language understood by the patient and his family. The bond between patient, his family, and the physician is greatly strengthened by personal, face-to-face discussions where words are supplemented by body language and where the patient and his relations have ample opportunities for clear lingering doubts. It is, at times, necessary to spread the process of informing the patient and his family over more than one session. This enables voicing of thoughts or anxieties experienced by the patient well after the doctor left his room.

Honesty remains the best policy in doctor-patient relations here as in any other situation. This is especially so when surgery is indicated. The expected query from the patient: "Are you sure that all will be well after the operation?" must elicit from the physician a succinct and reasoned account of current physical handicaps consequent to the damage already suffered by the medullospinal junction. The fact that handicaps due to permanent damage to tracts and neurons are likely to persist after treatment must be presented gently but firmly. This unwelcome information can be balanced by pointing out that treatment will prevent further deterioration that is otherwise inevitable. While the physician should in no way lay himself open to the charge of scare-mongering, realism must prevail. This is especially so when the patient or the family reject recommended therapy. Failure to impress on them the consequences of neglect of the disease also amounts to depriving them of information on which they can base their decision on proposed treatment.

As long as the physician is sincere in ensuring that not only is there no intention to harm the patient in any way but that all suggestions regarding treatment are made in order to promote recovery and prevent further handicaps, the patient and his family will have no cause for ill-feeling or complaint.

It is also important to make certain that the very best of treatment available under the circumstances is offered. There is no room for false pride or the urge to benefit from the patient's illness. Should the treatment be beyond the capability of the treating physician, the patient must be referred to another with proven expertise. An example is the patient who needs complex decompression of the medullospinal junction (perhaps via anterior and posterior routes) followed by fixation of the occiput and atlas to the subsequent vertebrae. There is no shame in acknowledging one's limitations and respecting the greater competence of a colleague in the interests of the patient.

Justice demands that all patients be treated only on the basis of the medical and surgical considerations that apply in their respective cases. Just as disease does not respect affluence or poverty, so should our treatment.

The patient's right to confidentiality demands that all discussions on his illness with others be carried out only after obtaining his express permission for them. Most patients prefer to have near relations to be with them when decisions – such as that on surgery – have to be made. Here, the patient's permission is implied by his having them invited to the exchange of ideas. (Also see the note above on medical records and conditions under which confidentiality may have to be breached.)

## THE ASYMPTOMATIC PATIENT

The patient with medullo-spinal compression who manifests neither symptoms nor signs poses special problems.

Clinical wisdom born of experience will inform the physician of the likelihood of the patient remaining asymptomatic. In this event, the task is simpler. It is necessary to inform the patient of his ailment, emphasizing at the same time that as yet it has produced no clinically detectable sequelae. The need for special care in order to ensure that there is no injury to the craniovertebral region must then be explained. Such injury may produce sudden weakness of limbs or other signs. Where required (as for instance in mobile dislocations of the atlas), the use of a collar can be prescribed.

Since handicaps from medullospinal compression may be irreversible or only partly reversible by surgery, we need to tell the patient about surgery as a possible permanent solution. The fact that surgery is intended to prevent neurological disorder should be emphasized.

The need to discuss preventive surgery is especially relevant when the patient has traveled a long distance to reach the clinic and may find return to the clinic, when symptomatic, difficult on account of costs or lack of facilities.

## THE PATIENT IN EXTREMIS

From time to time, a patient is brought into hospital, quadriplegic and out of breath. Tests show severe permanent damage to the medullospinal junction caused by long-standing compression. The thickness of the cervical cord at the level of the atlas may be reduced to that of a ribbon.

The prognosis is grim despite all medical care and decompressive surgery.

While one can consider offering the well-to-do patient all possible care, including artificial ventilation and surgery, care must be exercised before undertaking similar measures in the indigent.

The former patient will be able to afford the means for prolonged care, rehabilitative measures, round-the-clock nursing, and care of his urinary bladder.

The indigent patient's family may be destroyed should – as is most likely – the patient remain quadriplegic after surgery. In such families, dependent on daily wages and already struggling to make ends meet, the imposition of a long-term burden in the form of care for the crippled patient may tip the balance against them. As for the patient, inevitably, pressure ulcers will appear and deepen. Cystitis, nephritis, and pneumonia will follow. The patient will linger in misery till merciful death ends his tragic existence.

As the draft guidelines of Committee for Ethics and Medico-Legal Affairs of the World Federation of Neurosurgical Societies so well phrase it: "We must avoid serving as the surgeon of last resort for a desperate patient seeking surgical intervention that has been rejected by many of our peers and that may not help the patient or stop the advance of disease."

When in doubt, cogitate over whether it is better *not* to use a ventilator when the prognosis is hopeless. Clough's reworking of the Ten Commandments has provided us the basis for such a decision: "Thou shalt not kill; but need'st not strive officiously to keep alive.." [5] It is less difficult to justify not using a ventilator than it is to defend removal of a ventilator once it is in use.

The management of the patient with a bad prognosis may be less thorny in countries where the Living Will and Advance Directives by patients are recognized by law, provided the patient has documented his directions to his medical attendants and family while in full possession of his senses.

In countries – such as India – where such documentation is not recognized by the law, great care must be exercised in order to keep the patient's family informed of the patient's condition and hopeless outlook in order to enable them to make informed decisions. It is important to ensure that the medical team continues to do everything possible to relieve pain and distress in the patient and provide emotional support to the family.

## THE PATIENT WHO REFUSES RECOMMENDED SURGERY

Understanding and sympathy, not arrogance and abruptness, must govern the physician's approach in such instances.

Every attempt must be made to find out why the patient and his family have chosen to avoid surgery.

If fright be the cause, reasoning may help. Without minimizing risks and possible complications, the physician can juxtapose them against the likelihood of further harm to an already damaged medullospinal junction by continued and worsening compression.

If lack of funds is what holds the patient back, he can be reassured that the physician and his team will make every effort to help by calling upon the social service department and aiding the patient seek funds from public trusts and other charitable institutions.

If a blank wall exists between patient and physician despite all such measures, time may prove the great healer. Allow the patient to return home, assuring him that should he change his mind, he can seek help at any time and will receive the very best of help at the hospital.

It is unethical and cruel to berate patient and family for spurning well-meant scientific advice, refuse to provide medical documentation, and cast them off adrift because of their decision against surgery.

## THE VERY POOR PATIENT

Treating a patient can be difficult when he is abjectly poor, illiterate, and desperate enough to literally throw himself at your feet. Restoring his self-respect is our first task. Empathy rather than sympathy is in order. Patience worthy of Job [6] (".Ye have heard of the patience of Job.…" - King James' Bible), repeated and continued explanations of the arguments for and against the proposed treatment, must form the mainstay of our approach. Our account of likely complications must inform but not frighten the patient.

The patient may need help in meeting expenses within the hospital and outside it. Family members who have accompanied the patient to hospital, all of them traveling scores and even a hundred kilometers in the process, may also need sustenance. The hospital social service department can be of immense help here.

## CAUTIONARY NOTE

Do not equate the poor and illiterate patient with an unintelligent person. More often than not, this patient is blessed with more than average intelligence, sharpened by the need to survive under great handicaps and adverse circumstances.

## DETERIORATION IN NEUROLOGICAL STATE AFTER TREATMENT

Surgery on the craniovertebral junction is risky. In the decade 1950-1960, when excision of the posterior rim of the foramen magnum and of the posterior arch of the atlas formed the mainstay of treatment, a few patients at the Sir Jamsetjee Jejeebhoy Group of Hospitals in Byculla and King Edward Memorial Hospital in Parel, both in Bombay, India (as the city was named then), died from postoperative bleeding into the cervico-medullary junction. [7] Better anesthesia and instruments, and improved surgical techniques have prevented similar tragedy since.

Even so, from time to time we do face worsening in neurological condition – often short-lived.

If the patient and family have been informed of the likelihood of such an event, they will be prepared to face it.

When the family sees untiring efforts by doctors and other hospital staff on behalf of their patient and the latter experiences their concern and constant vigil, their anxiety is tempered by the fact that everything possible is being done.

Efforts made by the physician and his team in keeping the patient and his family informed of the progress or deterioration in neurological status and other developments is more than repaid by the trust engendered in the team's capabilities and understanding of the team's efforts and concern.

A strong rapport between patient, family, and the medical team can help avoid rough weather or storms after clouds have gathered around the patient.

In the event of the patient's demise, it is important that the most senior member of the medical team conveys the sad news to the family, summarizing once again the all-out efforts made to reverse the complications as and when they developed. Heart-felt condolences and a continuation of the offer to help at this stage – as during earlier stages – will go a long way in assuaging the grief felt by the family.

## IMPLANTS

The World Federation of Neurosurgical Societies (2008) includes in its guidelines on ethics a section entitled *Financial Concerns and Business Relationships*. In this section are the following recommendations:

"As neurosurgeons we should ideally limit the source of our professional income to services actually rendered to the patients under our supervision, for services we are personally and identifiably responsible to provide or oversee."

"Fees and terms for payment should be clearly communicated to patients, and they should be allowed a reasonable amount of time for payment without harassment. To the greatest extent possible, we should consider the financial resources of our patients, and we should treat patients as we would wish our families to be treated."

"In cases where the physician has a financial interest in an enterprise related to a patient's care, the patient should be informed of this fact."

"Material incentives to use any institution, service, medication, or equipment should never be accepted. Payments to encourage patient referrals should never be made."

These need emphasis. The use of implants, especially in orthopaedic surgery, has grown to an extent that huge monetary interests have come into play. The competition among the big players among those manufacturing implants has spawned unwelcome practices in which some surgeons are willing partners. These practices are to the detriment of patients and are, therefore, unequivocally unethical.

A measure of the extent to which the rot has set in can be gained from a recent news report by Sherman. [8] "Late last year (November 2007), the five top orthopedic device makers settled a U.S. Justice Department investigation over gift and payment practices the agency said were aimed at influencing surgeons' implant choices."

Four of them -- Zimmer Holdings Inc, Johnson and Johnson's DuPuy Orthopedics, Smith and Nephew and Biomet Inc -- agreed to pay a combined $311 million as part of the settlement. Zimmer paid the lion's share, $169.5 million.

"The companies also agreed to reforms, including federal monitoring.'

A conflict of interest exists when a surgeon is paid by the manufacturer of an implant to travel to centers abroad and use its products while extolling its virtues. The interests of the patient are now secondary to the surgeon's monetary and other benefits from the manufacturer.

Surgery on the craniovertebral region does not require these very expensive implants but it is important to ensure that those used are of the best quality and offered to the patient at the lowest available cost. Failure of plates, screws, rings, and other devices in the craniovertebral region can lead to severe morbidity or death.

## RELEVANT RESEARCH

In northern India, especially in the state of Uttar Pradesh, we encounter an unusually large number of congenital atlanto-axial dislocations. [9] While those seeking medical advice are being treated in local and distant hospitals throughout the country, it is essential to uncover the exact incidence of such dislocations and the cause for this high prevalence.

Surveys, especially in hamlets and small towns, will provide invaluable data. Such surveys must include clinical evaluations for such features as short neck, webbed shoulders, torticollis, and neurological abnormalities and for plain x-ray evaluation of such individuals and of samples of the population showing no abnormality. It may be necessary to incorporate other methods of study such as genetic evaluation, identification of abnormal nutrition or metabolism, and the presence of toxins in food or water.

Clusters of other varieties of craniovertebral abnormalities in other parts of India and in other poor countries may need similar study.

Should one or more causes be identified, it may be possible to devise preventive measures with the immeasurable benefits that will follow.

## SUMMING UP

Most patients have similar requirements. They want a doctor who is fully competent to deal with all aspects of their illness. Following close upon this need is that for consideration, kindness, and courtesy. The latter demands that the doctor treat the patient as a human being endowed with intelligence, curiosity about his illness, and fear of outcome. If we listen to what the patient has to say, not merely at the first meeting but on each occasion, we gain insights into his anxieties and his strengths. Building on the latter, we can allay – to the extent that is honestly possible – the former. Explanations in simple language, offered repeatedly if required, go a long way in reassuring the patient and his family.

If we respect the patient, remain true to his best interests, and do our best for him, we shall have more than met his expectations.

The Golden Rule is a great help (UNESCO report). [10] If I put myself in the position of the patient and ask myself the question: "What would I expect of my physician?" and act on the basis of these expectations, I am unlikely to go wrong.

Polonius' recommendation is cast in the same mould: "This above all: to thine own self be true, And it must follow, as the night the day, Thou canst not then be false to any man." (*Hamlet*, act 1, scene. 3, lines 78-80). [11]

I commend a study of the detailed guidelines on ethics in neurosurgery by the World Federation of Neurosurgical Societies.
